# Indocyanine Green Near-Infrared Fluorescence-Guided Sentinel Lymph Node Biopsy in Colon Cancer

**DOI:** 10.3390/biomedicines13040902

**Published:** 2025-04-08

**Authors:** Vlad Fagarasan, Vasile V. Bintintan, Radu I. Seicean, Giorgiana Fagarasan, David Andras, Emil Botan, Gabriel Samasca, George C. Dindelegan, Calin I. Cainap

**Affiliations:** 1 1st Surgical Department, “Iuliu Hațieganu” University of Medicine and Pharmacy, 400012 Cluj Napoca, Romania; vlad.fagarasan@yahoo.com (V.F.); rseicean@yahoo.com (R.I.S.); dr.andrasdavid@gmail.com (D.A.); george.dindelegan@gmail.com (G.C.D.); 2 Department of Anatomy and Embriology, “Iuliu Hațieganu” University of Medicine and Pharmacy, 400012 Cluj-Napoca, Romania; giorgianaamarinei@yahoo.com; 3 Department of Pathology, County Emergency Clinical Hospital, 400347 Cluj-Napoca, Romania; emilbotan@gmail.com; 4 Department of Immunology, Iuliu Hatieganu University of Medicine and Pharmacy, 400006 Cluj-Napoca, Romania; gabriel.samasca@umfcluj.ro; 5 Department of Medical Oncology, “Iuliu Hațieganu” University of Medicine and Pharmacy, 400012 Cluj-Napoca, Romania; calincainap2015@gmail.com

**Keywords:** indocyanine green, sentinel lymph node, lymphatic mapping, colon cancer

## Abstract

**Background/Objectives:** Indocyanine green (ICG)-guided near-infrared (NIR) fluorescence imaging represents a potentially advantageous approach for the identification of lymphatic drainage pathways. This study was undertaken to evaluate the efficacy of ICG-guided NIR fluorescence in mapping lymphatic drainage and facilitating sentinel lymph node biopsy (SLNB) in patients diagnosed with colon cancer. **Methods:** A prospective cohort of 30 consecutive patients with colon cancer undergoing surgical resection at our institution was enrolled in this study. Peritumoral injection of ICG was performed to facilitate intraoperative identification of sentinel lymph nodes (SLNs). Identified SLNs were marked and excised ex vivo following specimen retrieval. All the retrieved specimens were submitted for histopathological analysis using hematoxylin and eosin (H&E) staining. SLNs that were negative for metastatic disease upon H&E staining underwent further examination via immunohistochemistry (IHC). **Results:** Successful identification of SLNs was achieved in 83.33% of cases. The false positive rate was 6.6%, and the false negative rate was 8%, respectively. Atypical lymphatic drainage patterns were observed in 6.6% of the patients. Notably, the patients exhibiting atypical lymphatic drainage subsequently developed metastases during the follow-up period. Immunohistochemical analysis failed to detect micrometastases in SLNs that were initially deemed negative based on H&E staining. **Conclusions:** NIR–ICG fluorescence is a safe, reliable, and technically feasible method for performing SLNB in patients with colon cancer. Furthermore, this technique offers the potential for intraoperative identification of atypical lymphatic drainage pathways, which may have significant implications for determining the optimal extent of standard lymphadenectomy.

## 1. Introduction

Colorectal cancer remains a significant global health concern, ranking as the third most commonly diagnosed malignancy in both males and females. Moreover, it represents the second leading cause of cancer-related mortality, accounting for approximately 9.6% of all cancer diagnoses. While mortality rates have shown a decline, the worldwide incidence of colon cancer continues to rise [1]. In patients diagnosed with non-metastatic colon cancer, the presence and extent of lymph node metastasis serve as a critical prognostic indicator. Furthermore, lymph node status functions as an independent prognostic factor following curative resection of colonic malignancies [2].

The landscape of neoadjuvant treatment strategies for colon cancer is constantly evolving, leading to increasingly complex decision-making processes for both patients and healthcare professionals. The current standard of care involves upfront surgical resection followed by adjuvant chemotherapy, typically recommended for patients diagnosed with stage III disease [3]. This recommendation is often extended to stage II colorectal cancer patients exhibiting adverse prognostic factors, including large tumor size, insufficient lymph node evaluation, perineural or lymphovascular invasion, poorly differentiated tumor grade, intestinal obstruction, tumor perforation, or high-grade tumor budding [4]. While chemotherapy is not universally recommended for all stage II patients due to its associated toxicities, a significant proportion (approximately 10–20%) subsequently experience locoregional recurrence or distant metastasis [5]. Analysis of the SEER database has revealed that stage II colon cancer, particularly stages IIB and IIC, can exhibit a poorer prognosis compared to stage IIIA, potentially due to inaccurate staging [6]. This discrepancy may stem from the underestimation of the disease burden in patients with lymph node metastasis resulting from aberrant lymphatic drainage patterns or diagnostic inaccuracies.

Histopathological analysis of excised tissues using hematoxylin-eosin (H&E) staining remains the standard diagnostic method for detecting lymph node metastasis. However, H&E staining exhibits limitations in identifying micrometastases (MM) and isolated tumor cells (ITC), leading to potential underestimation of disease burden. Studies have indicated that a substantial proportion (20–30%) of colorectal cancer patients initially classified as lymph node-negative based on H&E staining demonstrate positive findings upon subsequent immunohistochemical analysis [7]. Consequently, the advancement and implementation of supplementary analytical techniques to enhance the detection of lymph node metastasis is warranted [8]. Given the considerable volume of lymph nodes typically excised during colon cancer surgery, the widespread application of immunohistochemistry to each node presents significant logistical and financial challenges. Therefore, prioritizing the identification of specific targets for immunohistochemical analysis, such as sentinel lymph nodes (SLNs), is crucial for optimizing resource allocation and improving diagnostic accuracy.

Sentinel lymph node biopsy (SLNB) has established itself as a dependable method for predicting nodal status in breast cancer and melanoma [9,10]. While numerous studies have validated its efficacy, SLNB in colorectal cancer has primarily served to enhance the accuracy of nodal staging. This cautious approach stems from concerns regarding the inconsistent lymphatic drainage patterns characteristic of the colon [11,12]. Various techniques facilitate SLN detection, including radiocolloids, colored dyes, and fluorescent compounds like indocyanine green (ICG) [7,11,12,13]. ICG has demonstrated improved detection rates and increased accuracy in identifying sentinel lymph nodes in patients with breast cancer, compared to previously described techniques such as blue dye or radiocolloid injection [14]. We hypothesize that these advantages have the potential to translate in patients with colon cancer and offer the possibility of upstaging patients who would otherwise be considered node-negative. Early investigations into SLN detection in colon cancer yielded suboptimal results, potentially attributable to the use of blue dye and radioactive tracers [15]. However, ICG has emerged as a more promising agent for SLN identification in colon cancer patients, demonstrating high accuracy and detection rates, with particularly high sensitivity for T1-T2 tumors [16]. However, despite the potential advantages of this technique, issues concerning increased false negative rates and decreased sensitivity have been reported [13]. Concerns regarding poor visualization due to a thickened mesentery in obese patients or obstruction of the lymphatic ducts in patients with advanced tumors have prevented the widespread application of this technique.

The present study aims to evaluate the clinical utility of near-infrared indocyanine green (NIR–ICG)-guided SLN biopsy in patients with colon cancer by determining the accuracy of SLN detection. A secondary objective involves investigating whether immunohistochemical analysis of the identified SLNs offers incremental benefits compared to standard H&E staining for histopathological assessment.

## 2. Materials and Methods

### 2.1. Inclusion and Exclusion Criteria

This prospective, observational, longitudinal cohort study investigated patients diagnosed with and treated for colon cancer at the 1st Surgical Department of the County Emergency Clinical Hospital of Cluj Napoca, Romania, between March 2022 and July 2024. Ethical approval was obtained from the Bioethics Committee (Reference DEP68/11.03.2022) prior to the commencement of the study, and informed consent was secured from all the participating patients upon admission for their planned surgical intervention. The study included patients over the age of 18 years diagnosed with clinical stage I or II colon cancer who underwent primary surgical treatment. Exclusion criteria encompassed patients with stage III or metastatic colon cancer, synchronous malignant tumors, bowel obstruction, or cancer of the appendix or recto-sigmoid junction.

### 2.2. Patient Preparation

Prior to surgical intervention, all the patients underwent a colonoscopy with biopsy of the colonic lesion. Histopathological analysis of the biopsied tissue was performed to confirm the presence of malignant cells. Furthermore, the patients received a comprehensive computed tomography (CT) scan of the head, chest, abdomen, and pelvis to determine loco-regional staging and to rule out the presence of metastatic disease. Standard preoperative assessments also included routine blood tests and quantitative analysis of the tumor markers carcinoembryonic antigen (CEA) and CA 19-9. Preoperative bowel preparation consisted of a mild regimen using Lactulose (30 mL) administered three times daily for three days before surgery. Anticoagulant therapy was administered the evening before surgery, and antibioprophylaxis was administered at the time of incision. Intraoperatively, compression stockings were utilized for deep vein thrombosis prophylaxis. Enhanced Recovery After Surgery (ERAS) perioperative protocols were implemented, incorporating peridural anesthesia, early mobilization, early enteral feeding, limited use of intra-abdominal drains, intraoperative removal of the nasogastric tube, and a restriction on postoperative opioid analgesia.

### 2.3. Sentinel Lymph Node Identification and Biopsy

**Figure 1:**
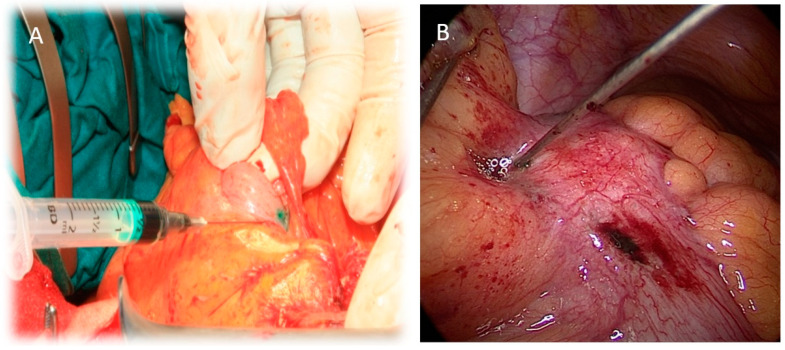
The administration of ICG solution during surgical procedures. Panel (**A**) depicts the injection technique employed during open surgery, while Panel (**B**) shows the ICG injection procedure in a laparoscopic surgical setting. These contrasting approaches highlight the adaptability of ICG imaging across different surgical modalities.

To facilitate the identification of SLNs and the lymphatic drainage pathways in the colon, ICG was employed as a tracer. A stock solution of ICG was prepared by reconstituting one vial of Verdye (5 mg/mL) with 10 mL of sterile water. This initial solution was further diluted to a final concentration of 0.5 mg/mL. Subserosal injections of the diluted ICG solution were then administered at four cardinal points around the target area on the colon. Each injection site received a volume of 0.25 mL. For laparoscopic procedures, a modified injection technique was utilized to minimize the risk of tumor cell contamination of the abdominal wall. An 18-gauge needle, serving as a trocar, was inserted, and a 26-gauge spinal needle was passed through the 18-gauge needle to perform the subserosal ICG injections. A visual representation of the injection methodologies employed in both open and laparoscopic surgical approaches is provided in Figure 1.

**Figure 2:**
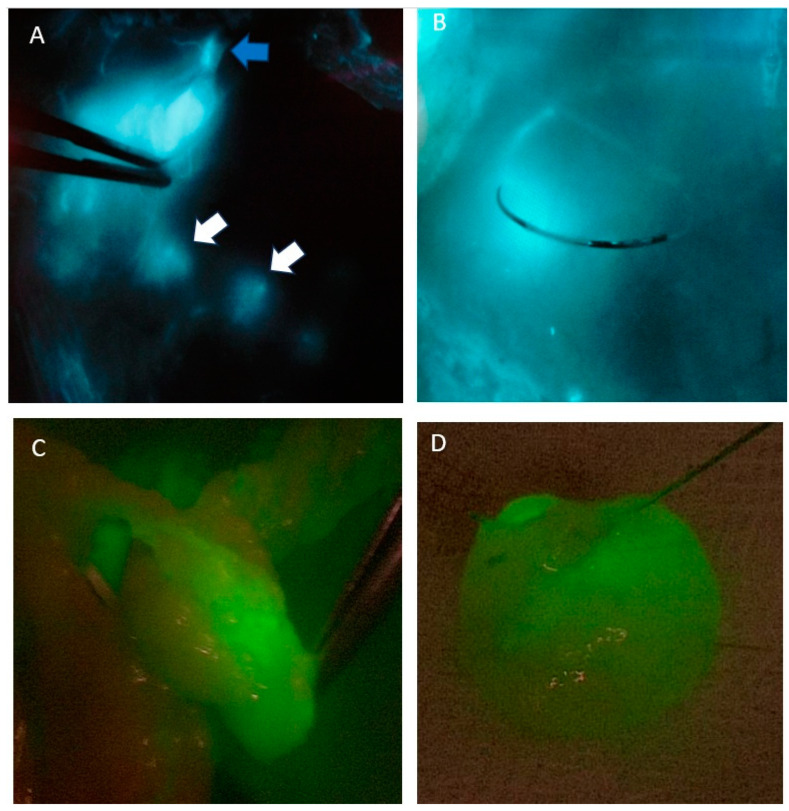
The SLNB procedure (**A**) demonstrates the detection of SLN, where the blue arrow denotes the injection site of the tracer and the white arrows indicate the identified SLNs; (**B**) displays the intraoperative marking of a SLN with a surgical suture to facilitate its identification and subsequent removal; (**C**) depicts the ex vivo harvesting of the identified SLN; while (**D**) presents the excised SLN specimen following removal from the patient.

ICG detection was performed intraoperatively using the SPY Portable Handheld Imaging System (Stryker) for open surgical cases or the IMAGE1 S Rubina system (Karl Storz) for laparoscopic procedures. Following the ICG injection, lymphatic mapping was initiated, with SLNs identified within 5 to 8 min. Lymph nodes visualized beyond this timeframe were not classified as SLNs. However, the presence of ICG within lymphatic vessels outside the typical lymphatic drainage pathways was documented as a separate finding due to its potential clinical significance. To maintain oncological integrity, SLNs were harvested ex vivo after the surgical specimen was removed from the patient. All the patients underwent a standard colon resection with complete mesocolic excision (CME). The process of SLN identification and subsequent biopsy is visually represented in Figure 2.

The histopathological analysis of the excised specimens adhered to standard protocols for colon tumors as defined by the College of American Pathologists (CAP) [17]. Separately retrieved SLN specimens were submitted for histopathological evaluation using H&E staining. SLNs exhibiting negative results upon H&E staining underwent further immunohistochemical analysis. This secondary analysis employed pancytokeratin AE1/AE3 antibodies, following a previously established methodology for SLN detection [18]. The cases in which lymphatic tissue was identified on histopathological analysis were considered true positive cases. The false positive percentage was defined as the proportion of cases in which the fluorescent tracer was identified intraoperatively but the histopathological analysis did not confirm the presence of lymphatic tissue on the excised specimen. The false negative percentage was defined as the proportion of cases in which lymph node biopsies were performed, but the identified lymph nodes were not sentinel lymph nodes.

### 2.4. Statistical Analysis

The data retrieved from the hospital database for this case series included patient demographics (age and sex), the American Society of Anesthesiologists (ASA) score, tumor location, surgical approach, and preoperative tumor staging according to the Union for International Cancer Control (UICC) TNM classification. Furthermore, information regarding tumor size, degree of differentiation, postoperative TNM staging (based on histopathological analysis), the total number of excised lymph nodes, the number of lymph nodes exhibiting metastasis, and length of postoperative hospitalization was collected. Postoperative complications were classified according to the Clavien–Dindo classification system [19]. Patients were followed prospectively for up to two years postoperatively through routine outpatient consultations. Tumor recurrence, both local and distant, was diagnosed via Positron Emission Tomography–Computed Tomography (PET–CT) scans performed during the oncological follow-up period.

The statistical analysis was conducted utilizing GraphPad Prism version 9.3.0 (GraphPad Software, San Diego, CA, USA). Descriptive statistics were employed to calculate means and percentages for continuous variables. Categorical variables were analyzed using contingency tables. The Spearman correlation coefficient was utilized to quantify the degree of association between independent variables, with the resulting analysis presented in a correlation matrix. Survival analysis was performed using the Kaplan–Meier method, with comparisons between survival groups assessed via the log-rank test, and hazard ratios with 95% confidence intervals. Overall survival (OS) was defined as the duration from the initial diagnosis of the colon tumor at the index colonoscopy to the date of the patient’s death. Disease-free survival (DFS) was defined as the interval between surgical intervention and the diagnosis of disease recurrence, or until the patient’s death. A significance level of *p* < 0.05 was adopted for all the statistical tests.

## 3. Results

A total of 256 patients underwent surgery for colon cancer in our clinic during the specified time period. Following the acquisition of informed consent, a cohort of 30 patients met the inclusion criteria and were enrolled in the present study. A comprehensive summary of the demographic profiles and tumor-specific pathological characteristics of these participants is detailed in Table 1.

**Figure 3:**
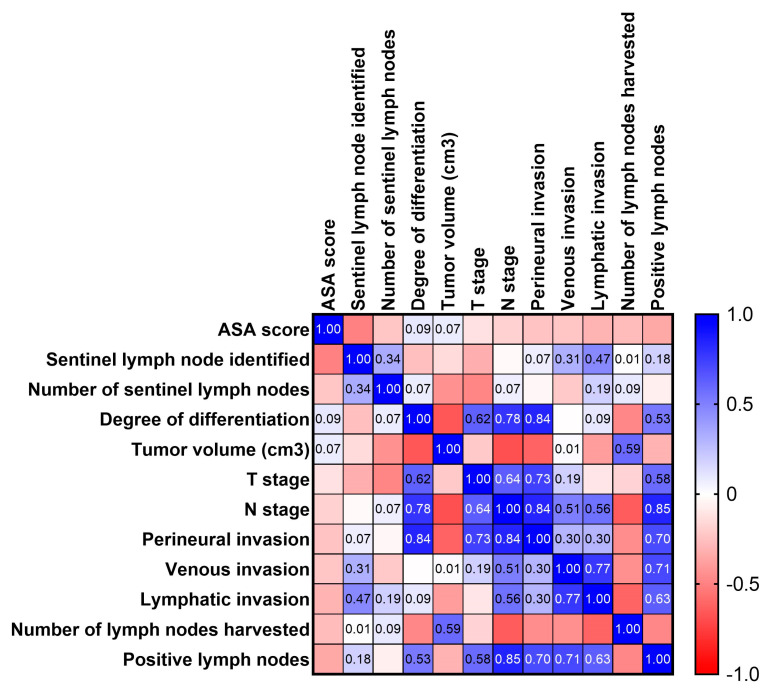
Correlation matrix illustrating the relationships between tumor−specific characteristics and the rate of SLN detection.

The study cohort had a mean age of 67.53 years (95% CI 63.49–71.58), with ages ranging from 41 to 86 years. The majority of tumors (60%) were located in the left colon, while five cases presented with tumors at the splenic flexure and were categorized as transverse colon tumors. The mean tumor volume was 34.85 cm^3^ (95% CI 1.03–68.66), with a range of 0.04 cm^3^ to 480 cm^3^. While initial imaging studies indicated node-negative disease in all the cases, postoperative histopathological analysis using H&E staining revealed lymphatic metastasis in seven patients. Tumor deposits in proximity to the primary tumor were observed in two patients (6.66%), classifying them as pN1c. In these cases, ICG fluorescence was not recognized on the tumor deposits during surgery. The mean number of harvested lymph nodes was 17.1 (95% CI 13.11–21.09), and the mean number of positive lymph nodes was 1.07 (95% CI 0.06–2.08). Statistical analysis revealed a significant association between patient age and ASA score, as well as between aggressive tumor characteristics (perineural invasion and tumor differentiation) and tumor stage according to the TNM classification. No significant correlation was found between the rate of SLN detection and tumor characteristics. The correlation matrix for all the investigated parameters is presented in Figure 3.

**Figure 4:**
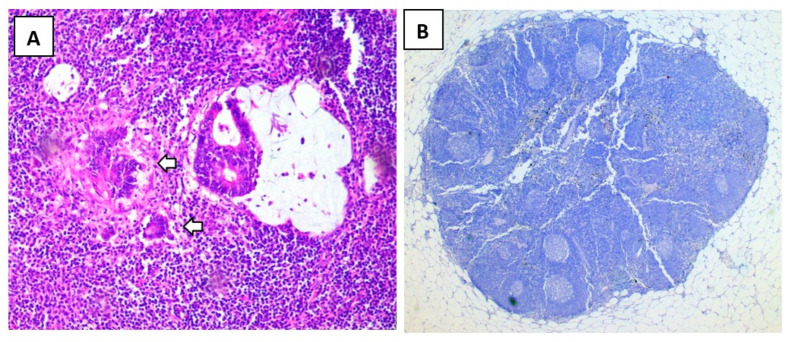
Histopathological analysis of resected lymph nodes. Panel (**A**) illustrates a conventional hematoxylin and eosin (H&E) stained section, magnified 10 times, revealing the presence of micrometastases indicated by white arrows. Panel (**B**) shows a section of a sentinel lymph node that tested negative, stained with pancytokeratin AE1/AE3, and magnified 2.5 times. This staining technique aids in visualizing epithelial cells and confirming the absence of metastasis in the examined sentinel lymph node.

Intraoperative SLN identification was successful in 25 out of 30 cases (83.33%). Histopathological analysis confirmed SLN status in 23 of these cases, with a false-negative rate of 8%. The false-positive percentage in the series was 6.6%. Two cases in the series demonstrated skip metastasis, having negative sentinel lymph nodes but positive non-sentinel lymph nodes: two of five positive lymph nodes in the first case, and one of four positive lymph nodes in the second case. The mean number of SLNs identified was 1.53 (95% CI 0.9809–2.086). In the remaining four cases where SLNs were not identified, elevated Body Mass Index (BMI > 30 kg/m^2^) was observed in four patients. One patient presented with a large primary tumor (pT4). Histopathological analysis in two cases revealed lymphatic vessels without lymph node structures. Furthermore, immunohistochemical analysis using pancytokeratin staining on harvested SLNs, initially deemed node-negative via H&E staining, was also negative in all cases. Representative images of the histopathological findings are presented in Figure 4. Atypical lymphatic drainage was observed in two cases (6.66%): one instance involved a para-aortic lymph node in station 216-b1, and the other involved an external left iliac artery lymph node in station 293L, based on the Japanese Classification of Colorectal Carcinoma [20]. In both cases, the primary tumors were located in the sigmoid colon. The atypical lymph nodes were dissected separately, and the harvested nodes were negative in both cases.

Postoperative morbidity was observed in eight cases (26.66%). Notably, no adverse events were associated with the injection of the ICG tracer. Two patients experienced chylous ascites, which were managed via a fat-restricted diet and parenteral nutrition (Clavien–Dindo grade II) or radiological drainage of the chylous peritoneal fluid (Clavien–Dindo grade IIIa). An anastomotic leak developed in one patient, necessitating surgical re-intervention and the creation of a terminal colostomy (Clavien–Dindo grade IIIb). Prolonged postoperative ileus occurred in one patient, which was managed conservatively (Clavien–Dindo grade II), and a single patient experienced an acute psychiatric event. Acute urinary retention requiring intermittent urinary catheterization (Clavien–Dindo grade II) and hematochezia managed conservatively (Clavien–Dindo grade II) were each observed in one patient. Finally, one patient developed a severe COVID-19 respiratory infection with multiple organ dysfunction, requiring intensive care unit (ICU) management (Clavien–Dindo grade IVb). The mean length of hospitalization was 9.53 days (95% confidence interval: 7.896–11.17). No patient required readmission within the first 30 days post-discharge.

**Figure 5:**
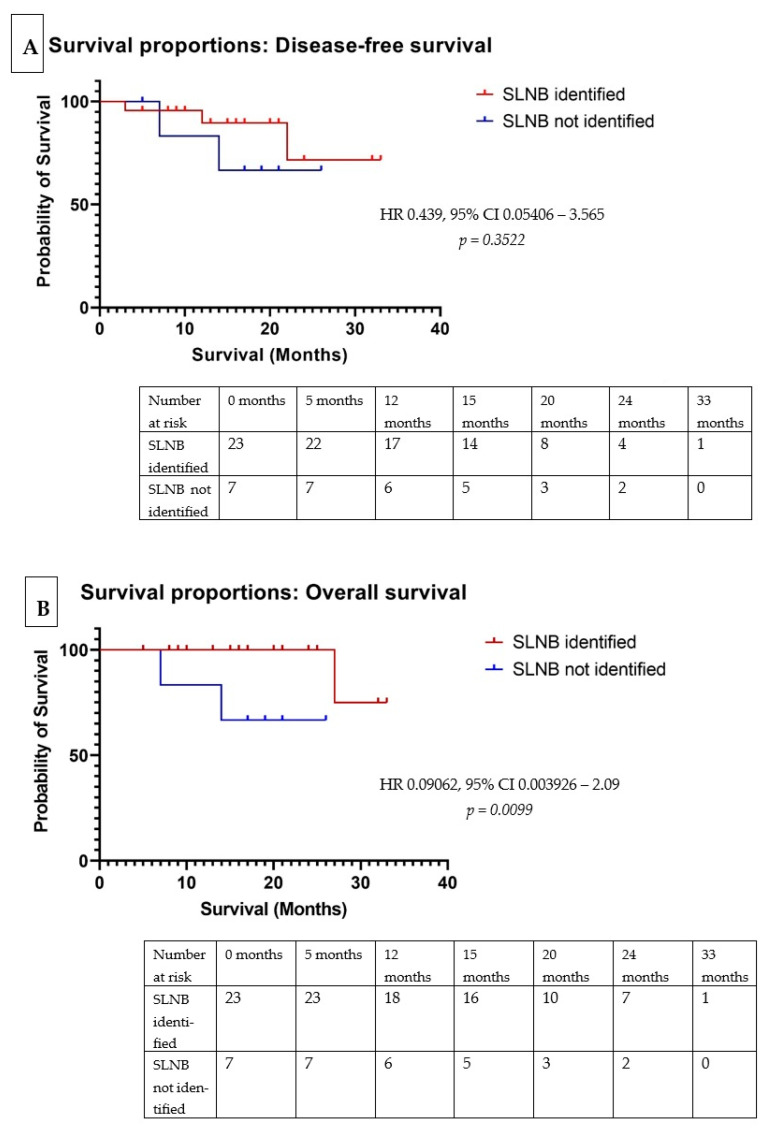
Kaplan–Meier survival analysis evaluating the impact of lymph node detection on patient outcomes within the studied cohort. Panel (**A**) illustrates DFS, while Panel (**B**) depicts OS. These analyses aim to determine the prognostic significance of lymph node involvement on the duration of time before disease recurrence and the length of survival from diagnosis in this patient population.

The OS rate for the entire cohort was 96.29% at one year and 91.77% at two years of follow-up. DFS rates were 88.52% at one year and 69.88% at two years. No local recurrences were observed. Three patients experienced distant recurrence: two developed hepatic metastases within 18 months of follow-up, specifically in cases exhibiting atypical lymphatic drainage, while one patient presented with cerebral metastasis at 12 months post-operation. A subgroup analysis comparing patients with successful SLN identification to those without revealed a statistically significant difference in OS (log-rank test, *p* = 0.0099, HR 0.09062, 95% CI 0.003926–2.093). However, no statistically significant difference in DFS was observed between these groups (log-rank test, *p* = 0.3522, HR 0.439, 95% CI 0.05406–3.565). The survival analysis results are detailed in Figure 5.

## 4. Discussion

Lymph node status remains a crucial prognostic indicator for predicting tumor recurrence and survival in patients diagnosed with locally advanced colorectal cancer [21]. Accurate assessment of lymph nodes within resected specimens is fundamental to the current staging system for this disease and is also essential for identifying patients who may benefit from adjuvant chemotherapy following surgical intervention. While SLN biopsy has been successfully adopted into clinical guidelines for breast cancer and melanoma, its routine application in colorectal cancer is not widely recommended due to concerns about the sensitivity of the technique in detecting macrometastases [7,22,23]. In the present study, the overall detection rate was 83.33%, and the accuracy in identifying SLNs reached 92%. This accuracy surpasses that reported in studies utilizing methylene blue as a tracer dye and aligns with findings from previous investigations that employed ICG as a tracer [15,24,25,26].

Near-infrared (NIR) light’s limited tissue penetration depth (approximately 5–7 mm) presents a potential challenge for SLN detection, particularly in obese patients [27]. This limitation is corroborated by our findings, where a thickened mesocolon accounted for over half of the cases where SLN identification failed. Bembenek et al. [7] reported SLN detection accuracy rates below 80% in obese patients and suggested a BMI threshold of less than 24 to improve accuracy and reduce false-negative rates. Another significant limitation arises from advanced tumors, which can obstruct lymphatic channels with tumor cells. Although statistical analysis in this study did not reveal a significant correlation between T stage/tumor volume and successful SLN identification, the relatively small sample size may have impacted this result. Nevertheless, the presence of advanced tumors could explain the observed survival differences between the study groups.

In the present study, immunohistochemical analysis of H&E-negative lymph nodes failed to reveal additional MM or ITC. Pancytokeratin AE1/AE3 antibodies, consistent with established SLN examination protocols for various tumor types [17,18], were employed for IHC. While previous research by Wang et al. [28] demonstrated that IHC using this methodology can result in upstaging in up to 12.5% of node-negative colon cancer patients, our findings did not replicate this result. Furthermore, the potential for increased tumor cell detection in H&E-negative nodes through the implementation of supplementary IHC protocols utilizing antibodies such as anti-p53, anti-VEGF-C, or anti-CD34 has been suggested [29]. The discrepancy between our findings and previous studies may be attributed to the meticulous evaluation of H&E-stained SLNs conducted by the pathologist, leading to a higher rate of micrometastasis identification compared to standard pathological analysis. This rigorous initial assessment may have minimized the potential for further detection via IHC.

SLN mapping utilizing indocyanine green fluorescence offers the added advantage of identifying aberrant lymphatic drainage pathways. Bilchik et al. [30] posited that SLN biopsy could detect such aberrant drainage in a notable proportion of patients with digestive tumors. This detection could potentially influence the extent of lymphadenectomy, thereby mitigating the risk of locoregional recurrence. A recently published study by Sun et al. [31] demonstrated that ICG lymphatic mapping could potentially improve the completeness of laparoscopic periaortic lymph node dissection in patients with left-sided colorectal cancer. In the present study, we observed lymph nodes located outside the typical lymphatic basin in 6.66% of cases. This incidence exceeds previously reported rates, which range from 1% to 40% with a mean detection rate of 5.1% [7,32,33]. Interim analysis of the GREENLIGHT trial [34] suggests that the prevalence of atypical lymphatic drainage may be substantially higher, approaching 41.4%, and that ICG lymphatic mapping provides clinically relevant information that could enhance the accuracy of curative resections. However, the clinical significance of these findings remains to be definitively established in the literature. Prior investigations have indicated that stage III pN1 colon cancer patients exhibiting skip metastasis demonstrate a higher incidence of hepatic recurrence and poorer overall and recurrence-free survival compared to patients without skip metastases [35]. Notably, the two cases in our cohort that presented with atypical lymphatic drainage patterns developed liver metastases within the initial 18 months of follow-up. In both instances, SLNs, as well as additionally excised lymph nodes, were negative upon both immunohistochemical and standard H&E analysis and thus categorized as N0. Further research investigating the impact of atypical lymphatic drainage patterns on survival outcomes in node-negative colon cancer patients is warranted to deepen our understanding of this infrequent observation.

The study’s principal strength lies in its standardized methodology. We present a reproducible method of subserosal ICG injection for laparoscopic colon resections, requiring no highly specialized equipment and applicable to a broad patient population. However, the technique exhibits limited applicability in morbidly obese patients due to abdominal wall thickness and in cases where the tumor is located near the rectosigmoid junction. The study’s main limitations include a relatively small sample size, precluding robust statistical analysis, and the chosen subserosal injection method. The latter presents specific intraoperative challenges, notably tracer spillage and consequent fluorescent dye contamination of the operative field, potentially hindering SLN identification. Despite these limitations, this study offers a valuable overview of our experience with NIR–ICG fluorescence for in vivo SLN biopsy in colon cancer patients, suggesting it is a feasible and safe technique.

## 5. Conclusions

NIR imaging with ICG fluorescence represents a safe and viable technique for SLNB in patients undergoing resection for colon cancer. Studies have demonstrated a high detection rate using this technique in both open and laparoscopic procedures. Furthermore, NIR–ICG fluorescence imaging offers the potential for intraoperative identification of aberrant lymphatic drainage patterns, which may necessitate modifications to the standard extent of lymphadenectomy.

## Figures and Tables

**Table 1:** Demographic and tumor-specific characteristics of the cohort.

Variable	Number of Cases (Percentage)
Sex	
Male	19 (63.33%)
Female	11 (36.66%)
ASA score	
2	13 (43.33%)
3	13 (43.44%)
4	4 (13.33%)
Tumor location	
Cecum	2 (6.66%)
Ascending colon	3 (10%)
Transverse colon	7 (23.33%)
Descending colon	6 (20%)
Sigmoid colon	12 (40%)
cT stage	
Tis	6 (20%)
T1	4 (13.33%)
T2	6 (20%)
T3	12 (40%)
T4	2 (6.66%)
Surgical approach	
Open	14 (46.66%)
Laparoscopic	16 (53.33%)
pN stage	
N0	22 (73.33%)
N1	6 (20%)
N2	2 (6.66%)
Lymphatic invasion	
L0	24 (80%)
L1	6 (20%)
Venous invasion	
V0	22 (73.33%)
V1	8 (26.66%)
Perineural invasion	
Pn0	21 (70%)
Pn1	9 (30%)

## Data Availability

The raw data supporting the conclusions of this article will be made available by the first author on request.

## References

[1] Bray F., Laversanne M., Sung H., Ferlay J., Siegel R.L., Soerjomataram I., Jemal A. (2024). Global cancer statistics 2022: GLOBOCAN estimates of incidence and mortality worldwide for 36 cancers in 185 countries. CA Cancer J. Clin..

[2] Akagi Y., Adachi Y., Ohchi T., Kinugasa T., Shirouzu K. (2013). Prognostic impact of lymphatic invasion of colorectal cancer: A single-center analysis of 1,616 patients over 24 years. Anticancer Res..

[3] Smith H.G., Nilsson P.J., Shogan B.D., Harji D., Gambacorta M.A., Romano A., Brandl A., Qvortrup C. (2024). Neoadjuvant treatment of colorectal cancer: Comprehensive review. BJS Open.

[4] Baxter N.N., Kennedy E.B., Bergsland E., Berlin J., George T.J., Gill S., Gold P.J., Hantel A., Jones L., Lieu C. (2022). Adjuvant Therapy for Stage II Colon Cancer: ASCO Guideline Update. J. Clin. Oncol..

[5] Nors J., Iversen L.H., Erichsen R., Gotschalck K.A., Andersen C.L. (2024). Incidence of Recurrence and Time to Recurrence in Stage I to III Colorectal Cancer: A Nationwide Danish Cohort Study. JAMA Oncol..

[6] Li Y., Hua R., He J., Zhang H. (2022). Survival Contradiction in Stage II, IIIA, And IIIB Colon Cancer: A Surveillance, Epidemiology, and End Result-Based Analysis. Evid. Based Complement. Alternat Med..

[7] Bembenek A., Schneider U., Gretschel S., Fischer J., Schlag P.M. (2005). Detection of lymph node micrometastases and isolated tumor cells in sentinel and nonsentinel lymph nodes of colon cancer patients. World J. Surg..

[8] Hamada K., Otsubo R., Takeshita H., Nonaka T., Tominaga T., Hidaka S., Sumida Y., Abe K., Sawai T., Nagayasu T. (2019). Diagnosis of Lymph Node Metastasis in Colorectal Cancer by a Semi-dry Dot-blot Method. Anticancer Res..

[9] Giammarile F., Vidal-Sicart S., Paez D., Pellet O., Enrique E.L., Mikhail-Lette M., Morozova O., Maria Camila N.M., Diana Ivonne R.S., Delgado Bolton R.C. (2022). Sentinel Lymph Node Methods in Breast Cancer. Semin. Nucl. Med..

[10] Phan G.Q., Messina J.L., Sondak V.K., Zager J.S. (2009). Sentinel lymph node biopsy for melanoma: Indications and rationale. Cancer Control.

[11] Stojanoski S., Manevska N., Antovic S., Pop-Gjorcheva D., Vaskova O., Miladinova D., Mileva M. (2017). Sentinel Lymph Node Detection in Colorectal Cancer—First Experience. Open Access Maced. J. Med. Sci..

[12] Saha S., Philimon B., Efeson M., Helina A., Elgamal M., Kiya G., Hilkiah S., Arora M., Wiese D., Kitagawa Y. (2022). The role of sentinel lymph node mapping in colon cancer: Detection of micro-metastasis, effect on survival, and driver of a paradigm shift in extent of colon resection. Clin. Exp. Metastasis.

[13] Currie A.C. (2019). Intraoperative Sentinel Node Mapping in the Colon: Potential and Pitfalls. Eur. Surg. Res..

[14] Wang Z., Cui Y., Zheng M., Ge H., Huang Y., Peng J., Xie H., Wang S. (2020). Comparison of indocyanine green fluorescence and methylene blue dye in the detection of sentinel lymph nodes in breast cancer. Gland. Surg..

[15] Des Guetz G., Uzzan B., Nicolas P., Cucherat M., de Mestier P., Morere J.F., Breau J.L., Perret G. (2007). Is sentinel lymph node mapping in colorectal cancer a future prognostic factor? A meta-analysis. World J. Surg..

[16] Burghgraef T.A., Zweep A.L., Sikkenk D.J., van der Pas M.H.G.M., Verheijen P.M., Consten E.C.J. (2021). In vivo sentinel lymph node identification using fluorescent tracer imaging in colon cancer: A systematic review and meta-analysis. Crit. Rev. Oncol. Hematol..

[17] Washington M.K., Berlin J., Branton P., Burgart L.J., Carter D.K., Fitzgibbons P.L., Halling K., Frankel W., Jessup J., Kakar S. (2009). Protocol for the examination of specimens from patients with primary carcinoma of the colon and rectum. Arch. Pathol. Lab. Med..

[18] Weaver D.L. (2010). Pathology evaluation of sentinel lymph nodes in breast cancer: Protocol recommendations and rationale. Mod. Pathol..

[19] Dindo D., Demartines N., Clavien P.A. (2004). Classification of surgical complications: A new proposal with evaluation in a cohort of 6336 patients and results of a survey. Ann. Surg..

[20] Japanese Society for Cancer of the Colon and Rectum (2019). Japanese Classification of Colorectal, Appendiceal, and Anal Carcinoma: The 3d English Edition [Secondary Publication]. J. Anus Rectum Colon.

[21] Kim H.J., Choi G.S. (2019). Clinical Implications of Lymph Node Metastasis in Colorectal Cancer: Current Status and Future Perspectives. Ann. Coloproctol..

[22] Chatterjee A., Serniak N., Czerniecki B.J. (2015). Sentinel lymph node biopsy in breast cancer: A work in progress. Cancer J..

[23] Brănişteanu D.E., Cozmin M., Porumb-Andrese E., Brănişteanu D., Toader M.P., Iosep D., Sinigur D., Brănişteanu C.I., Brănişteanu G., Porumb V. (2022). Sentinel Lymph Node Biopsy in Cutaneous Melanoma, a Clinical Point of View. Medicina.

[24] Staniloaie D., Budin C., Vasile D., Iancu G., Ilco A., Voiculescu D.I., Trandafir A.F., Ammar T., Suliman E., Suliman E. (2022). Role of methylene blue in detecting the sentinel lymph node in colorectal cancer: In vivo vs. ex vivo technique. Exp. Ther. Med..

[25] Guo H., Luo Y., Fu Z., Wang D. (2024). Indocyanine green fluorescence imaging for lymph node detection and long-term clinical outcomes in colorectal cancer surgery: A systematic review and meta-analysis. World J. Surg..

[26] Negrut R.L., Cote A., Feder B., Bodog F.D., Maghiar A.M. (2025). Enhanced Lymph Node Detection in Colon Cancer Using Indocyanine Green Fluorescence: A Systematic Review of Studies from 2020 Onwards. J. Pers. Med..

[27] Teraphongphom N., Kong C.S., Warram J.M., Rosenthal E.L. (2017). Specimen mapping in head and neck cancer using fluorescence imaging. Laryngoscope Investig. Otolaryngol..

[28] Wang F.L., Shen F., Wan D.S., Lu Z.H., Li L.R., Chen G., Wu X.J., Ding P.R., Kong L.H., Pan Z.Z. (2012). Ex vivo localization and immunohistochemical detection of sentinel lymph node micrometastasis in patients with colorectal cancer can upgrade tumor staging. Diagn. Pathol..

[29] Sfeclan M.C., Mirea C.S., Ciorbagiu M.C., Vîlcea A.M., Obleagă C.V., Pădureanu V., Cârţu D., Țenea-Cojan T.Ș., Moraru E., Vîlcea I.D. (2017). Immunohistochemical Study of Sentinel Lymph Node in Colon Cancer. Curr. Health Sci. J..

[30] Bilchik A.J., Saha S., Tsioulias G.J., Wood T.F., Morton D.L. (2001). Aberrant drainage and missed micrometastases: The value of lymphatic mapping and focused analysis of sentinel lymph nodes in gastrointestinal neoplasms. Ann. Surg. Oncol..

[31] Sun Y., Tang Z., Deng Y., Xu Z., Chen Z., Huang S., Wang X., Zheng Z., Lin H., Jiang W. (2024). Safety and efficacy of indocyanine green fluorescence imaging-guided laparoscopic para-aortic lymphadenectomy for left-sided colorectal cancer: Preliminary results of a case-matched study. Asian J. Surg..

[32] Chen M.Z., Tay Y.K., Prabhakaran S., Kong J.C. (2023). The management of clinically suspicious para-aortic lymph node metastasis in colorectal cancer: A systematic review. Asia Pac. J. Clin. Oncol..

[33] Lucas K., Melling N., Giannou A.D., Reeh M., Mann O., Hackert T., Izbicki J.R., Perez D., Grass J.K. (2023). Lymphatic Mapping in Colon Cancer Depending on Injection Time and Tracing Agent: A Systematic Review and Meta-Analysis of Prospective Designed Studies. Cancers.

[34] Ribero D., Mento F., Sega V., Lo Conte D., Mellano A., Spinoglio G. (2022). ICG-Guided Lymphadenectomy during Surgery for Colon and Rectal Cancer-Interim Analysis of the GREENLIGHT Trial. Biomedicines.

[35] Chang C.Y., Lin C.C., Lin H.H., Lan Y.T., Chang S.C., Wang H.S., Yang S.H., Chen W.S., Lin J.K., Jiang J.K. (2023). The Negative Prognostic Impact of Lymph Node Skip Metastasis in Stage III Colon Cancer With pN1 Disease: A Single-Center and Retrospective Cohort Study. Dis. Colon Rectum.

